# The Prevalence of Oral Mucosa Lesions in Pediatric Patients

**DOI:** 10.3390/ijerph191811277

**Published:** 2022-09-08

**Authors:** Joanna Elżbieta Owczarek-Drabińska, Patrycja Nowak, Małgorzata Zimoląg-Dydak, Małgorzata Radwan-Oczko

**Affiliations:** 1 Department of Oral Pathology, Wroclaw Medical University, 50-376 Wroclaw, Poland; 2 Student Scientific Society of Oral Health, Department of Oral Pathology Wroclaw Medical University, 50-376 Wroclaw, Poland

**Keywords:** oral mucosa lesions, children, prevalence, oral health

## Abstract

The prevalence of oral mucosa lesions (OMLs) among children varies from 4.1% up to 69.5%. There is a lack of sufficient epidemiological data and adequate knowledge about OMLs in relation to minors’ gender and age. The aim of the study was to evaluate the prevalence of OMLs in children, patients of the oral pathology clinic in south-western parts of Poland, and to investigate the potential correlation between the occurrence of particular types of OMLs and the gender and age. A retrospective study was performed using a total of 2474 clinical charts from 2015 to 2019. Data collected included age, gender, and OMLs’ type. The prevalence of OMLs in minors was 5.21%. Aphthae was the most frequent diagnosis. Boys were more commonly affected, and traumatic erosion and ulcers were significantly more often detected in males. The mean age of children was 8 y/o, preschoolers were significantly more often diagnosed with geographic tongue, while *Morsicatio buccarum* was significantly more common in school children and adolescents. Clinicians should be familiar with OMLs prevalence and with its specific frequency in connection to age and gender of children. Furthermore, they should be aware of the diversity of OMLs found in the oral cavity of children and that their frequency in the pediatric population is different from that in adults.

## 1. Introduction

Treatment of childhood caries and the consequences of tooth injuries are the main concerns of dental practitioners, which successfully overshadow other oral cavity pathologies observed in childhood [1,2,3], which are oral mucosa lesions (OMLs). The prevalence of OMLs in children is not that uncommon and rare as many clinicians may presume. Its incidence varies between research from 4.1% up to 69.5% [1,4]. This discrepancy in the results is influenced by the geographical provenience, development period of minors, and different methodological criteria used in the studies [1,2,4,5,6,7,8]. Nevertheless, oral mucosal conditions are underestimated and underdiagnosed not only by dentists, but also by pediatricians, dermatologists, and other medical specialists [1,2,4,5,9,10]. This can be disquieting when we realize that some of the OMLs can manifest and/or precede systemic diseases or disorders that can disturb child’s proper development or can be possibly life threatening [11,12]. Vitamins and trace elements deficiencies, autoimmune disorders (including Crohn’s disease, celiac, Behcet’s disease), hematological diseases, and fever syndromes are just a few among many maladies that can be reflected in the condition of oral mucosa. Ulcers and erosions, changes in the color and symmetry of the mucosa, depapillation of the tongue, and abnormal growth of the tissue are the signs and symptoms that should raise clinical suspicion. On the other hand, many OMLs are benign and self-limiting conditions that should not be treated—e.g., geographic or hairy tongue [11,12,13,14]. Therefore, the adequate knowledge of practitioners about the types and prevalence of OMLs in the pediatric population is essential for proper diagnosis, and hence appropriate therapy (or forbear from it). Nevertheless, the prevalence of oral mucosa diseases in the pediatric population is not of much interest in studies compared to the adult population [2,9,10,15]. This may be surprising when we realize that the types and symptoms of OMLs observed in children differ from those observed in adults or seniors [3,9,10]. On the other hand, most of the available literature on mucosal pathology in pediatrics focuses on mucosal disorders associated with oncology therapy and on subjects with specific chronic diseases [3,6,9,10]. The aim of the study was to evaluate the prevalence of OMLs in children aged 0–17 who were patients of the oral pathology clinic and to investigate the potential correlation between the occurrence of particular types of OMLs and the gender and age of the studied pediatric population.

## 2. Materials and Methods

A retrospective study of the oral cavity clinical charts of patients who referred to the oral pathology clinic in 2015–2019 was conducted. Oral cavity medical records were reviewed for the presence of oral mucosa lesions. A total of 2747 clinical charts were analyzed with the diagnosis of any type of oral mucosa abnormality. The patients came from the south-western part of Poland. The medical records were examined by a dentist, specialist in oral pathology, with the help of calibrated dentistry students. Relevant retrospective data such as the type of oral mucosa disease, age, and gender were collected. No other personal data was described or used. Diagnoses of the mucosal lesions found in the medical charts were made mainly on the basis of examination, observation, and clinical interview. Traumatic erosions and ulcers were most often caused by injuries (traumatic events or habits, e.g., biting or chewing the oral mucosa), unsuitable fillings, orthodontic appliances, food burns, or iatrogenic to the dental care. The lesions identified on the basis of a clinical examination and meticulous anamnesis were: aphthae, *Morsicatio buccarum*, hairy and geographic tongue, fibroma, abnormal upper lip frenulum, traumatic erosions and ulcers, gingival enlargement, melanoplakia, herpetic stomatitis, gingival cyst, ankyloglossia, frictional hyperkeratosis, eruptive cyst, *Linea alba*, granulomatous cheilitis, gingivitis, mucosal atrophy, pigmented nevus, leukoedema, Epstein pearls, Bohn nodules, bone exostosis, and exfoliative cheilitis. In order to confirm the clinical diagnoses of mucocele, fibroma, papilloma, pyogenic granuloma, Heck’s disease, a biopsy with full histopathology was performed. In addition, mycological examinations were always performed when oral candidiasis was suspected. If an allergic reaction was surmised as a trigger for OML, allergic blood tests followed by a consultation with an allergist were performed.

The inclusion criteria for the study were age from 1 day to 17 years, the diagnosis of OML confirmed in the medical records between years 2015–2019, and consent to participate in the study. Failure to meet any of the above criteria resulted in exclusion from the research. The informed, voluntary consent of all legal guardians of the children was obtained during a telephone conversation. The approval of the local Ethics Committee was not required due to the use of retrospective data and the inability to identify an individual person on the basis of data collected from the medical files.

The results of the obtained research were statistically analyzed. Data were presented as mean and standard deviation (SD). The verification of the hypothesis about the equality of the mean parameters in independent groups was carried out by the ANOVA method of variance analysis or for groups with heterogeneous variance with the nonparametric Mann–Whitney U test (for two groups) or the Kruskal–Wallis rank sum test (for a larger number of groups), and the homogeneity of variance was checked by Levene’s test. For discrete parameters, the frequency of the feature occurrence in groups was analyzed with the χ^2^_df_ test with the appropriate number of degrees of freedom df (df = (m − 1) × (n − 1), where m—number of rows and n—number of columns). *p* ≤ 0.05 was considered statistically significant. Statistical analysis was performed with EPIINFO Version 7.1.1.14, CDC, Atlanta, GA, USA and Statistica Version 13.3., TIBCO Software Inc. Palo Alto, Santa Clara, CA, USA.

## 3. Results

Out of all 2747 investigated charts, 143 belonged to minors, including 60 girls and 83 boys. That comprised of 5.21% of all of the outpatients admitted to the clinic between years 2015 and 2019. 3.28% of all of the females and 9.07% of all of the males admitted during this period. The mean age of all of the pediatric patients was around 8 years old, also in both genders. The average age of preschool children (in the entire study group, as well as girls and boys) was approximately 3 y/o. The mean age of school-age children in the entire group was 10 y/o, girls were slightly younger (9.6 y/o), and boys were slightly older (10.5 y/o). The average age of adolescents in the entire group, as well as in both sexes, was 15.5 y/o. (Table 1). In this group of 143 children, 31 different oral mucosa conditions were diagnosed (Table 2).

After statistical analysis, diseases occurring in at least 6 subjects (4.2% of the studied population) were selected for further study. This allowed for the identification of the ten most frequent OMLs, which accounted for 74.8% of the entire examined pediatric medical documentation (107 patients). Table 3 and Table 4 show the distribution of the 10 most frequently diagnosed oral conditions.

**Figure 1:**
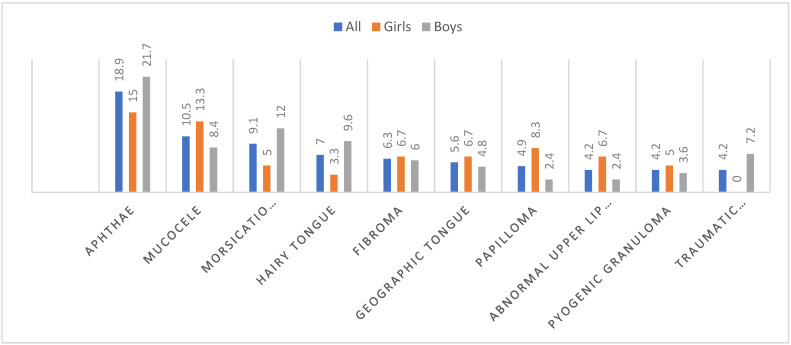
Distribution of the 10 most common OMLs by gender, in percentage.

The main reason for referral to the oral pathology outpatient clinic in the entire group was aphthae (18.9%), which was the most common in both girls and boys, 15% and 21.7%, respectively. The second most common diagnosis was mucocele (10.5%), which was also the second in girls (13.3%), but 4th in boys (8.4%). The third oral mucosa condition was *Morsicatio buccarum* (9.1%), which was the second most common complaint in boys (12%), but in girls it was one of the rarest lesions (5%). In female patients, the third most common problem was papilloma (8.3%), and in boys, a hairy tongue (9.6%). However, in these 10 most common diagnoses there was a difference in the distribution of OMLs between the sexes—only erosions and traumatic ulcers were significantly more often troubling boys than in girls (*p* = 0.03). More details are shown in Table 3, Figure 1.

For better understanding of the prevalence of OMLs in minors, the studied group of patients was divided into 3 age-dependent development groups: 0–6 years: preschoolers, 7–13 years: school-age children, and 14–17 years: adolescents [16].

**Figure 2:**
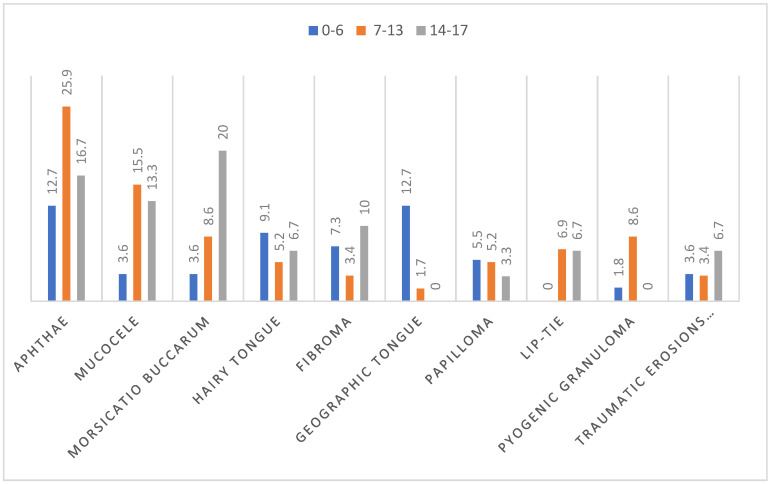
Distribution of the 10 most common OMLs by age groups, in percentage.

The frequency of certain OMLs varied with the age of our patients as well as in specific age-related developmental groups. In preschool children (0–6 years, mean age 2.82 y/o), who consisted of 38.5% of all of our patients, the most frequent oral mucosa alteration was equally aphthae and geographic tongue (12.7%), followed by a hairy tongue (9.1%) and fibroma (7.3%). In school children aged 7 to 13 y/o, mean age 10 y/o, (40.6%), aphthae was also the first reason for referral to the clinic (25.9%), the second was mucocele (15.5%), and the third pathology was equally shared between pyogenic granuloma and traumatic ulcers and erosions of the oral mucosa (8.6%). In our adolescent group (14–17 y/o, mean age 15.5 y/o), which was the least numerous (only 20.9% of the whole group), the most often diagnosis was *Morsicatio buccarum* (20%), followed by aphthae (16.7%) and mucocele (13.3%). Although the age distribution differed between the described age-related groups, only such OMLs as *Morsicatio buccarum* and geographic tongue revealed statistically significant age difference, *p* = 0.04 and *p* = 0.01, respectively. Geographic tongue was observed statistically more often in children aged 0–6 years, and *Morsicatio buccarum* was statistically more often diagnosed in school children and adolescents than in preschool children. All of the data are gathered in Table 4, Figure 2.

## 4. Discussion

Our study presents the prevalence and distribution of oral mucosa lesions in children and youths aged from 1 day until reaching the age of consent, which is 18 years. It should be emphasized, however, that this evaluation was performed in a group of patients with symptomatic oral mucosa lesions, who came to an oral pathology clinic with an existing problem. In our opinion, it is worth analyzing oral mucosa diseases that are diagnosed and treated in a specialist clinic, because such studies allow for the observation of the prevalence of particular diseases and the directions of their changes. Our work is focused on assessing the type, gender, and age-related distribution of oral mucosa lesions.

In the present study, the overall prevalence for clinically apparent oral mucosa lesions among pediatric patients was 5.21%. Similar incidence was reported by Kleinman et al. [17], Lima et al. [18], and Espinosa-Zapata et al. [19]: 4.08%, 6.6%, and 7.4%, respectively, although its higher frequency can be found, ranging from 9.73% up to 69.5% [1,2,3,4,9,20]. The discrepancy in pediatric OMLs incidence may be related to different research methodology, different nomenclature used to describe OMLs, variation in training, calibration, and examination features, also with different sampling frames (studies concern subjects from different geographical regions, ethnicities, cultures, and habits) [21].

**Figure 3:**
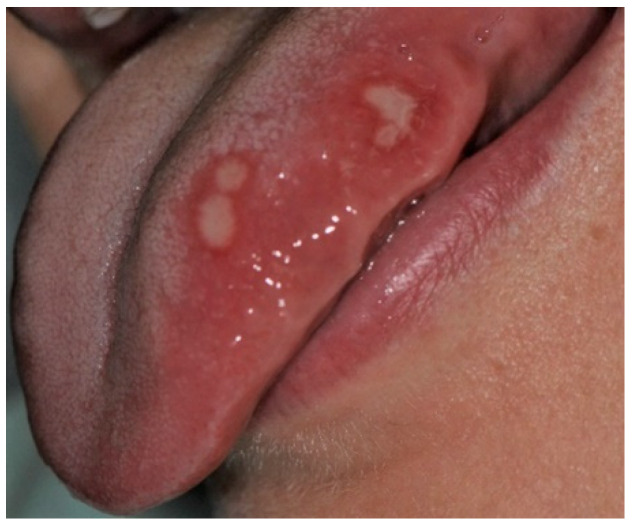
Minor aphthae on the left margin of the tongue in an adolescent boy. Four oval ulcerations with a yellowish-whitish removable fibrinopurulent membrane encircled by an erythematous halo.

The most prevalent mucosal pathology were aphthae (Figure 3) (18.9%), which were also the most frequently diagnosed in girls (15%) and boys (21.7%), and in preschoolers (12.7%, equally often with geographic tongue) and school-age children (25.9%). A similar overall incidence was reported by Vucicevic-Boras et al. among Croatian children aged 0–19, 14.8% [15]. Additionally, the current scooping review by Hong et al. [1] indicates aphthous ulcers the first, most prevalent OML among the world’s pediatric population, based on 57 reviewed studies and 85,976 cases in total. According to Shulman [2], recurrent aphthous stomatitis (RAS) frequency increases with age, which was confirmed by our findings. RAS were the most common oral mucosa disease in patients aged 0–6 and 7–13 years and the second most frequent in adolescents (16.7%), after *Morsicatio buccarum* (20%)

As aphthae are the most commonly diagnosed form of oral ulceration in childhood and adolescence, especially in the population with a high socio-economic status [1,2,3,8], practitioners should be aware that aphthous-like lesions may be a symptom of several entities, including inter alia: drug-induced mucocutaneous syndromes (e.g., due to use of proprionic acid, phenyloacetic acid, diclofenac, piroxicam, or betablockers), autoimmune diseases (e.g., Crohn’s and Behcet’s disease, celiac), hematologic disorders (e.g., anemia, neutropenia), fever syndromes (cyclic neutropenia, PFAPA (periodic fever, aphthous stomatitis, pharyngitis, cervical adenitis), Sweet syndrome), viral infections (e.g., HIV, COVID-19), or concomitant nutritional deficiencies [11,12]. Bearing this in mind, differential diagnosis process should always be performed prior to posing a final lesion recognition. In about 20% of patients having episodes of aphthae, haematinic deficiencies have been found. Notably, the deficiencies of vit. B12, folate and iron, occurring either alone or together, were positively associated with the aphthae development. Additionally, the low level of vit. B1, B2, and B6 have been reported in aphthous patients. RAS episodes were reported in 20% of patients with Crohn’s disease and in 10–26% of patients having coeliac or gluten intolerance. What is important and should be emphasized is that oral mucosa ulcerations may antedate the development of fully symptomatic disease by several years or decades. A similar pattern where 99% of patients present oral aphthae in any form can be observed in patients diagnosed with Behcet’s disease (a multisystemic disorder consisting of oral ulcerations and additionally any two of: skin and genital ulcers, eye lesions (uveitis, hypopyon, iridocyclitis) and positive result of pathergy test). Aphthous ulceration in the mouth can be an eminent symptom of anemia (aphthae observed in 34% of patients), cyclic neutropenia (bouts of fever every 21 days, lasting 3–5 days), or HIV-infected patients (severe, long-lasting aphthous lesions causing debilitating pain) [11,12]. Differential diagnosis may not always be easy as there is no definitive etiology or diagnostic/laboratory tests for aphthae, nor biopsy with full histopathology cannot provide the final interpretation. In this situation, identification of RAS is based on the combination of detailed medical history of the patient (and family), clinical signs and symptoms, blood testing, and eventually histopathology, which would reveal the non-specific inflammation process of the tissue [11,12]. Despite the quandary, a child with aphthae necessities a thorough workup in order to find an adequate diagnosis.

**Figure 4:**
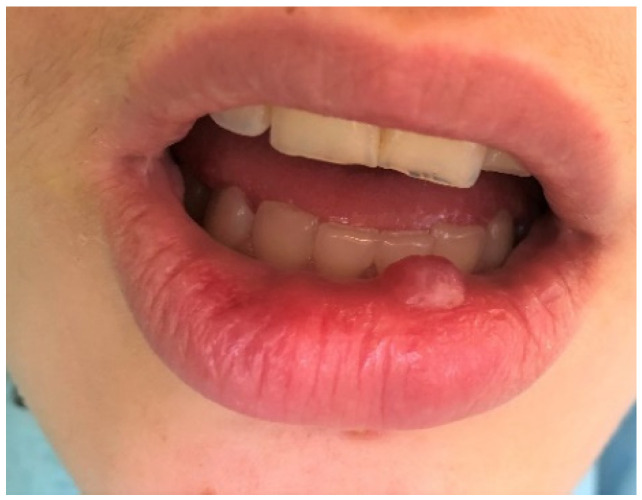
Mucocele on the lower lip of an adolescent girl. Typical dome-shaped fluctuant mucosal swelling. The lower lip is the most common site for this lesion to develop due to the relative ease of traumatizing this area.

Mucoceles are a common alteration of minor salivary glands due to trauma followed by obstruction of the salivary gland duct which effects cystic swelling, as seen in Figure 4 [22]. In this study they were diagnosed as the second most prevalent OML among the entire group of subjects (10.5%) among girls and school-age children. A similar incidence (13.5%) was revealed by Sousa et al. [23]. It was also the third most common lesion in adolescents, and one of the least frequent among preschool children (only two cases). This is not surprising, since many studies show that such disorders are often observed in the second decade of life and rarely in infants [24,25]. In our research, this pathology was more frequent among girls than boys, similar to observations made by Vale et al. [26]. In the same study, a histological revision of 315 diagnoses of pediatric patients revealed a very high incidence of mucocele, amounting to 33.3%. Other analyses by Wang et al. [27] and Lima et al. [18] also showed a high frequency of mucoceles, 24.5% and 17.2%, respectively. On the other hand, there are also other reports showing a lower prevalence of mucoceles in the pediatric population, but even in these studies with a lower incidence, mucoceles were always among the most commonly diagnosed OMLs [1,2,3,10,20,28,29,30].

**Figure 5:**
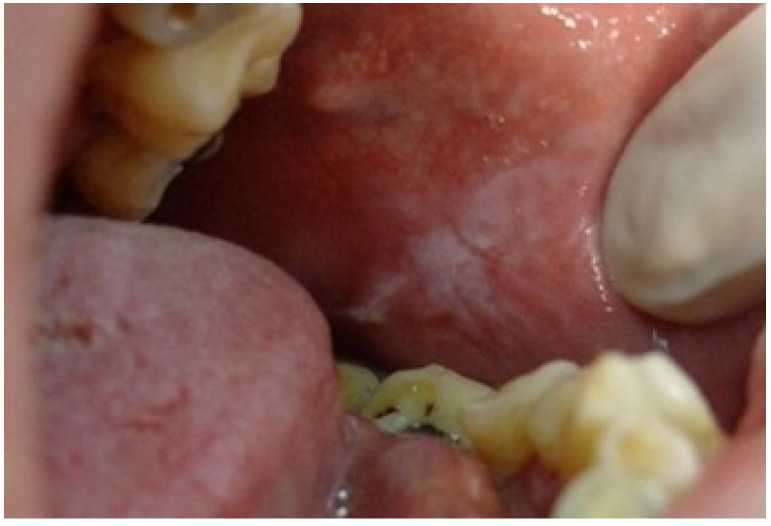
Morsicatio buccarum on the left buccal mucosa of a teenage boy. In this patient, the lesion was observed bilaterally in an atypical location in the midpart of the buccal mucosa along with occlusal line. The mucosa is white, thickened, shredded, and combined with zones of the erythema and erosion.

The third most frequently observed mucosal lesion in studied population was *Morsicatio buccarum* (MB). As pictured in Figure 5, this condition is characterized by a rough, whitish, shredded oral mucosa and develops due to the parafunctional, habitual biting/chewing of the buccal mucosa. The overall frequency of its occurrence was 9.1%, similar to what was observed among 0–12 y/o Brazilian children (8.4%) [27]. Jimenez et al. also found high incidence of this disorder, amounting 15.87%, among Venezuelan children aged 2–17 [28]. In many other researches, *Morsicatio buccarum* was diagnosed as one of the most common OMLs in children, but with varying incidence [2,3,17]. Nevertheless, it should be underlined that in many cases MB was included in the group of trauma-associated lesions (along with erosions, ulcers, etc. caused due to injuries or dental and orthodontic treatment), so it is difficult to directly compare the results [1,9,10,20], however the incidence rate of such compiled diagnoses was also very high in each study group. In our investigation, MB was the second most frequent diagnosis among boys and the 5th most commonly recognized in females, but there was no significant difference between the genders. This correlation was also observed in the work of Jimenez-Palacios et al. [28]. The number of MB cases was significantly increasing with age (*p* = 0.04), which was also observed by other researchers [2,9,10,27,28]. This observation can be explained that MB is considered as a parafunctional activity that develops in response to higher psychological overload, higher stress and anxiety, especially the one related to school and studying stress, which intensifies with the age of pupils [9,29,30,31].

**Figure 6:**
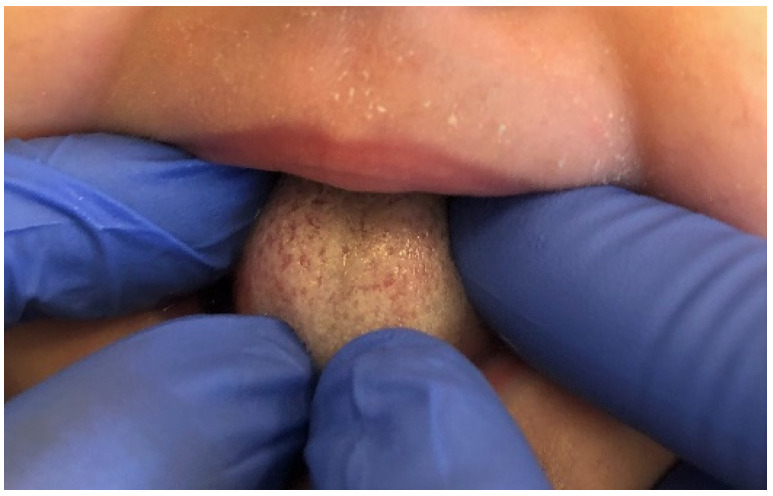
Lingua villosa on the dorsum of the tongue in the female infant. HT is characterized by elongated filiform papillae, especially in the midline and anterior part of the dorsum of the tongue.

The next most common diagnosis among pediatric patients who referred to the clinic was a hairy tongue (*Lingua villosa,* HT), 7%. The incidence of HT was lower than in the Spanish and Brazilian pediatric population, 16% and 23.4%, respectively [8,20]. However, our value was merely the same as that reported by Majorana et al. among Italian children (7.2%) [10]. Additionally, Hong et al. classified *lingua villosa* as one of the most commonly detected OML in the pediatric world population (overall relative global frequency of 0.28%), and the most prevalent mucosal pathology in Americas region [1]. This tongue lesion got its name due to the presence of elongated filiform papillae on the dorsum of the tongue, which gives a hairy-like appearance, as can be observed in Figure 6 [13]. In our investigation it was the 3rd most frequent OML in boys and one of the least in girls, 9.6% and 3.3%, respectively. However, no statistical difference between sexes or between age-related groups was found, as in other studies [8,10,20]. However, the incidence of HT was the highest among preschool children (9.1%), which was corroborated by the findings of the Brazilian study [8], and then it was decreasing among school-aged children (5.2%), then rose among adolescent group to the level of 6.7%. The greater prevalence of *lingua villosa* among young children (0–6 years) may be the outcome of the increased consumption of pulpy food, reduced oral hygiene skills, and the intake of certain diet supplements, e.g., vitamin C [13].

**Figure 7:**
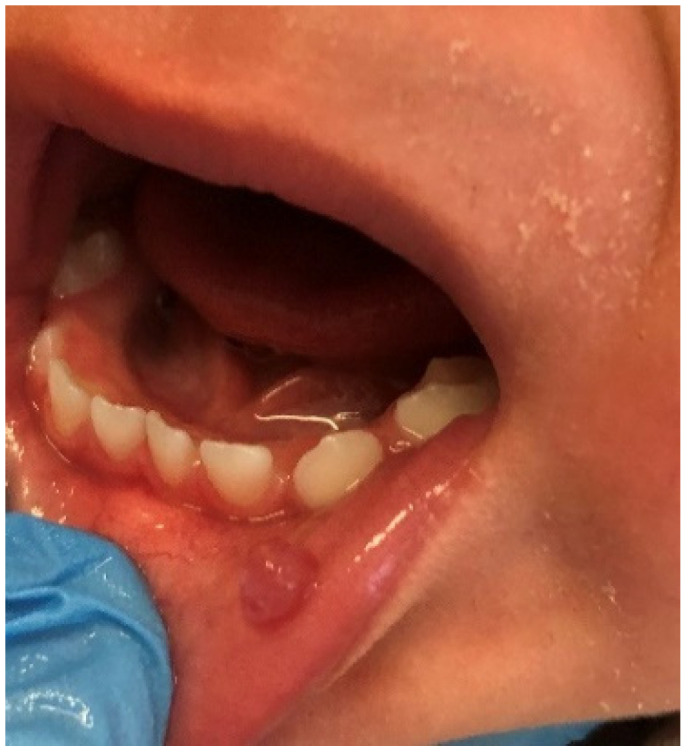
Fibroma on the lower lip of a female infant. A fibroma is a smooth, pink, dense nodule that arises in a chronically irritated area. The lesion has a broad peduncle and is painless and non-bleeding.

The prevalence of fibroma (Figure 7) was 6.3%. Additionally, it was at the same level between genders. Almost the same incidence value was reported by Wang et al.—6.14% [32]. However, in his study, the fibroma diagnosis rate decreased with age and was the highest in subjects aged 0–5 years and the lowest in 11–14 y/o. This was exactly the opposite of our findings, where the rate of fibroma among teenagers was the highest (10%). The incidence of fibroma varies between the studies from 0.01% up to 20.18% [1,2,3,33]. The variation in number is most probably explained by differences in the study design, including the sampling method (biopsies or clinical evaluation), age range, and geographic location of the study population. Generally, in cross-sectional studies, the incidence of fibroma is low, but when biopsied pathologies are taken into account, the frequency of this type of lesion increases dramatically from 4.23% (West Pacific) to 16.14% in Europe and 20.18% (Eastern Mediterranean region) [1,3]. In our study, for clinical recognition of fibroma, we always used both diagnostic methods, with clinical examination confirmed by histopathological evaluation.

**Figure 8:**
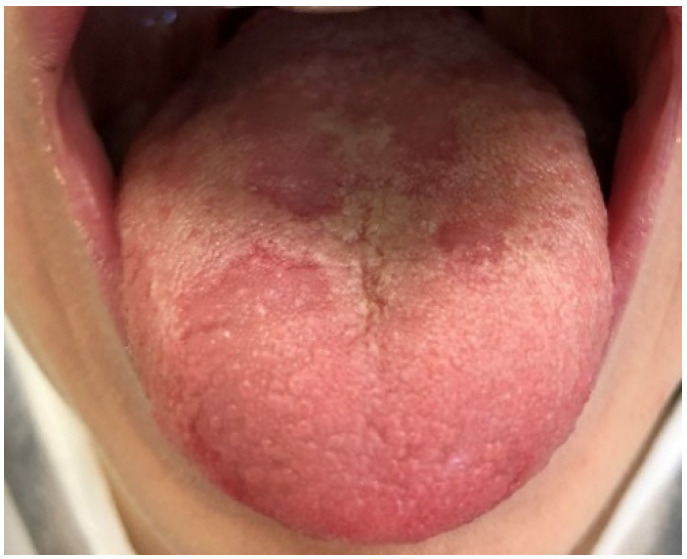
Geographic tongue (*Migratory glossitis*) in an 11-year-old boy. Characteristic, well-demarcated zones of erythema (atrophy of the filiform papillae) on the dorsal tongue mucosa, which may migrate over time to different sites of the tongue mucosa.

Geographic tongue (Figure 8) (*Migratory glossitis*) was found in 5.6% of the examined children, which was similar to the prevalence described in the study carried by Garcia-Pola et al. in Spain—4.48% [20]. Higher figures were detected in Brazil (9.1%) [27], Italy (almost 10%—two studies) [9,10], and Croatia (13.38%) [15]. Lower than the reported number on *Migratory glossitis* was observed in Argentina (3%), USA (1.08% and 0.6%) [2,17], and Turkey (1.72% and 0.8%) [7,34]. However, the global overall relative frequency is reported at a level of 1.29% [1]. In our study, there was no difference in the frequency of this pathology between genders, as it was also observed in previous researches [9,10,15,20,34]. However, its prevalence is changing with age. Preschoolers (aged 0–6 y/o) were significantly more often diagnosed with geographic tongue than school-aged children or adolescents (*p* = 0.01). Not a single case of this lesion was observed in adolescents in this research. Sedano et al. also revealed a strong difference between age groups in which children under ten years old were more prevalent to have *Migratory glossitis* [35]. Observations consistent with our findings were described by Majorana et al. in Italian children and by Vucicevic et al. in Croatian preschoolers, but not by Unur et al. and Amadori et al. Contrary to our study, their findings indicated that adolescents were more prone to be affected by this tongue lesion [9,10,15,34].

**Figure 9:**
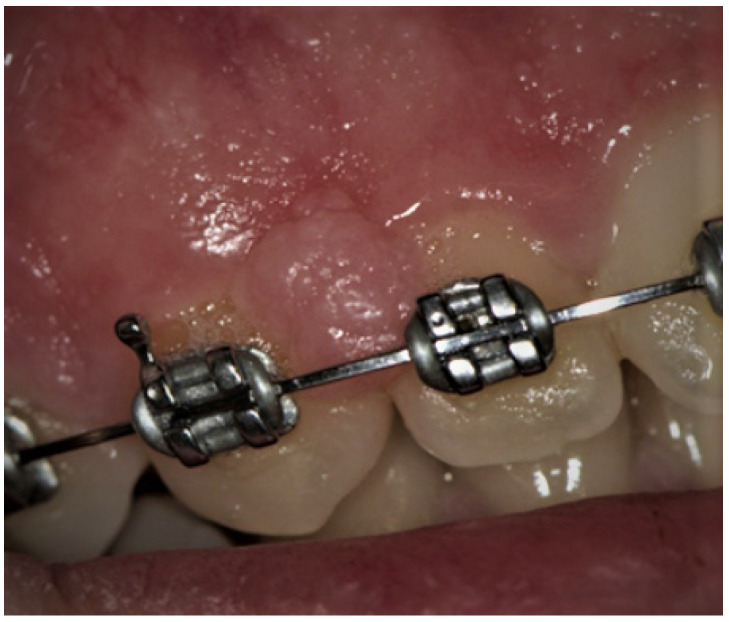
Papilloma of the interdental papilla between teeth 12 and 13 in adolescent girls. Papilloma is a soft, painless, pedunculated, exophytic nodule with uneven surface. Usually, it is a solitary lesion. In this patient, it has blunted projection and a pale-pink color.

In 4.9% of patients, papilloma was diagnosed (Figure 9). Wang et al. reported a similar rate—4.2%, Vale et al. slightly lower—3.8%, whereas studies from Northern Ireland and Turkey revealed its higher prevalence: 8% and 6.7%, respectively [26,32,33,36]. In studies where the diagnosis was based only on a clinical examination, the incidence of HPV infectious lesions was lower, ranging from 0.02% to 1.7% [2,9,10]. Additionally, the global frequency of papilloma diagnosed without biopsy, as presented in the scoping review by Hong et al., showed a fairly low incidence rate: 0.22%. However, in the same review, the overall prevalence of papilloma increased to 2.8% globally and 3.17% in Europe when specimen biopsy was used as a diagnostic criterion [1]. This difference may be related to the prevalence and frequency of Human Papilloma Virus subtypes, especially HPV 2,6,11 and 57, which are associated with the etiology of oral papilloma in different parts of the world. In the present study, HPV lesions were the third most common mucosal pathology observed in girls, and one of the rarest among boys, with almost equal distribution between age groups. The same relation between genders has been observed in previous reports [26,36]. In the studies by Wang et al. and Vale et al., being a teenager was considered a predictive factor of papilloma development. Research by Sato et al. showed the same prevalence of HPV oral lesions in different age groups, which corresponds with the findings of our investigation [37].

In the present study, the last three most common OMLs, with the same prevalence rate of 4.2%, were: pyogenic granuloma, traumatic erosions, and ulcers and overgrown upper lip frenulum.

**Figure 10:**
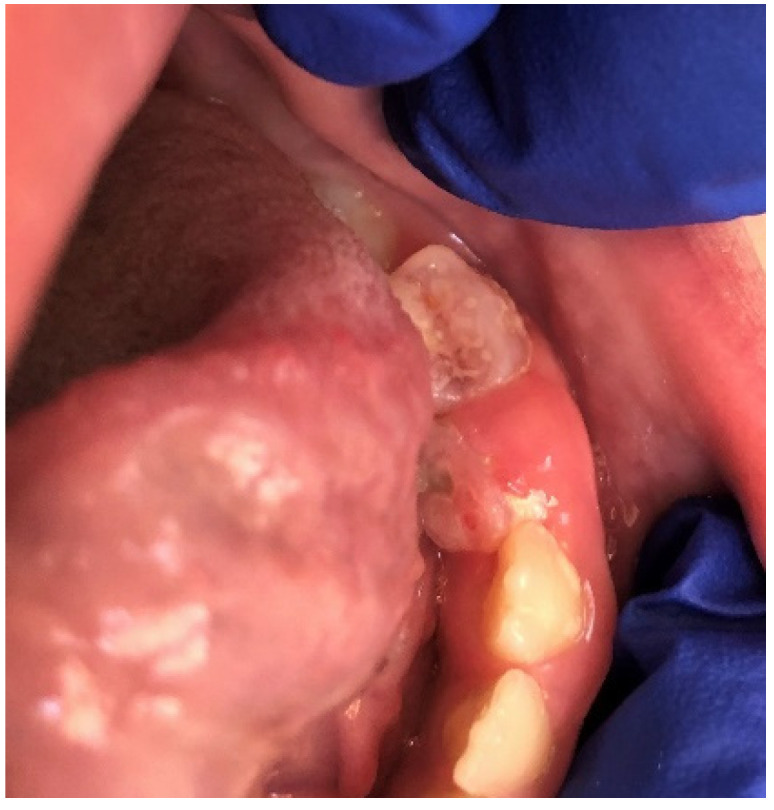
Pyogenic granuloma around 74 and 75 deciduous teeth in a school-aged female. In this case, pyogenic granuloma appears as a smooth, sessile mass with pink-reddish surface. Typically, the mass is painless and bleeds easily. Gingival irritation and inflammation due to poor oral hygiene—similar to this patient—may be a precipitating factor.

The incidence of pyogenic granuloma—4.2% (Figure 10)—was similar compared to the research carried out by Skinner et al. (4.1%) on 1525 patients aged 1–19 years [38]. This value was also close to the pooled global frequency (4.87%) presented in the most recent review, but lower than the European prevalence (8.82%) of this mucosal alteration revealed in the same article [1]. On the one hand, studies from the UK and Spain even showed a lower frequency than our figure or the European average, 3.06% and 2.2%, respectively [33,39]. On the other hand, Gultelkin et al., in her Turkey-based study, diagnosed pyogenic granuloma in 7% of all of her patients, and Sklavounou-Andrikopoulou et al. reported its incidence in a very high level of 35.1% in the Greek pediatric population [36,40]. In our study, children aged 7–13 and females were more frequently affected (the 5th most common diagnosis among girls, and 3rd among school-age children), which was also reflected in the previous findings [32,33,36,38].

**Figure 11:**
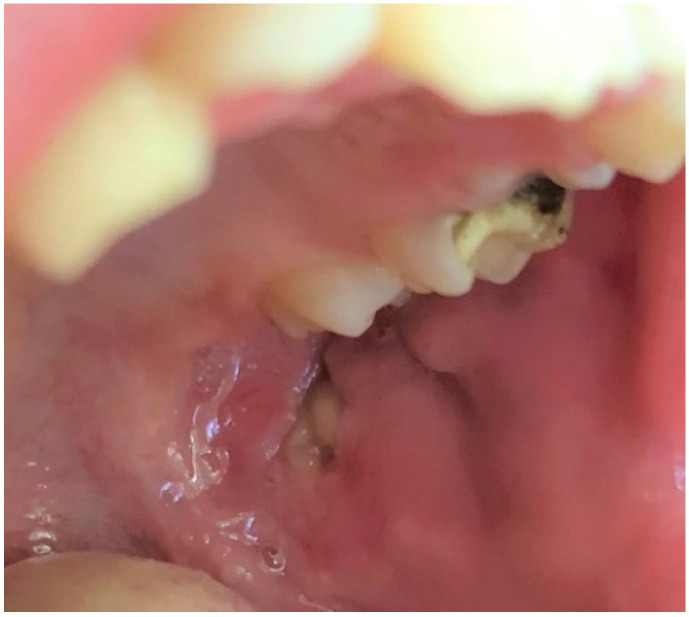
Traumatic ulcer on the left buccal mucosa in a school-age boy. Visible in the picture yellow-gray, oval, well-circumscribed ulceration with rolled border. In this case, the lesion resulted from a parafunctional chewing of the cheek due to school stress.

The prevalence of traumatic erosions and ulcers (Figure 11) among pediatric patients varies between studies, from as low as 1.33% up to 17.8%. This may be because the OMLs were classified differently, as certain studies included *Morsicatio buccarum* in this lesion category [8,9,10,15], while others, like the present study, did not [3,27,28]. In the investigations where MB was included in trauma induced mucosal lesions, its frequency was quite high, 11.8–17.8%, whereases when MB was considered as a separate pathology, the incidence of traumatic erosions and ulcers decreased to the level of 1.33% to 8.53%. In our opinion, it is more accurate to separate these two mucosal pathologies due to their different etiology, characteristics, and method of treatment. In our investigation, the prevalence of traumatic erosions and ulcers was equal in age groups, which confirmed the outcomes of the previous studies [15,27]. While other researchers did not find a gender predilection of this OML, we found a strong and significant correlation between traumatic erosions and ulcers and the male gender (*p* = 0.03) [8,27].

**Figure 12:**
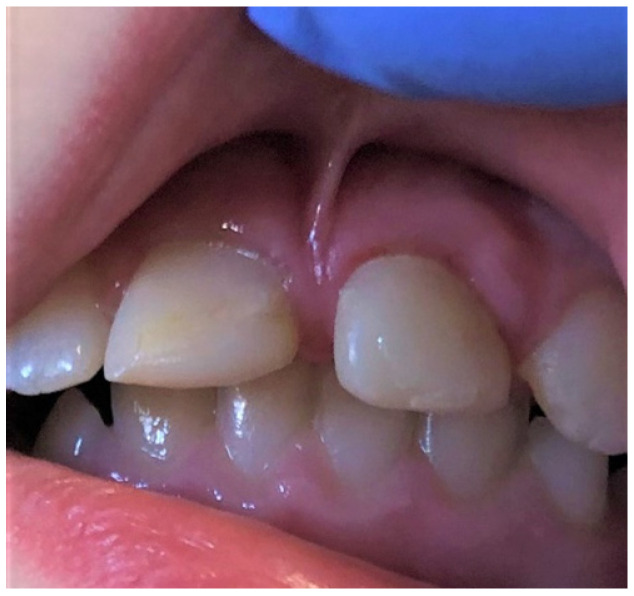
Overgrown, low, papilla penetrating attachment of the upper lip frenulum (type III acc. Placek) is causing a midline diastema in a school-age girl.

Overgrown or abnormal attachment of the upper lip frenulum may cause diastema, as can be seen in the case from Figure 12, promote the development of caries in the upper central incisors, especially in children, due to hindered tooth brushing, or predispose to periodontal problems [41]. The Placek classification of the upper labial frenulum regards papillary and papilla penetrating attachments as pathological (type III and IV) [42]. In our investigation frenulum attachments, type III or IV were diagnosed in 4.2% of all of the pediatric patients. This figure was lower than those presented by Thilander et al. (5–17 y/o Colombians), Bergese et al. (9–12 y/o Italians), and Kaimenyi (4–16 y/o Nigerian), 17%, 18.1%, and 35%, respectively. This difference may result from the method of selecting patients, as well as their ethnic origin [41,43,44,45]. Additionally, the above-mentioned studies used cross sectional sampling method, where our study was based on patients referring to an oral pathology clinic.

In the present study, overgrown or abnormal attachment of the upper lip frenulum was the 4th most common diagnosed problem in females (6.7%) and the least frequently in males (2.4%), which is in accordance with other investigations [41,43,44,45]. Approximately 7% of the abnormal upper lip frenulum was diagnosed in the age groups 7–13 y/o and in 14–17 y/o, with no cases in the youngest age group (0–6 y/o). For most of the children, 7 y/o is the time to start school, as well as the onset of mixed dentition. During this time, children are confronted with a larger group of peers and people, and the external appearance, including, of course, a smile, begins to play an import role. For some, diastema is a characteristic feature of a smile, and others may consider it a cosmetic defect that may lead to a disturbed self-esteem and start seeking help from a dental professional. This might be the reason for a higher frequency of patients aged 6–17 y/o referring to the clinic with the problem of overgrown or abnormal attachment of the upper lip frenulum.

Although this study provides helpful and important information on the sex and age-related incidence of oral mucosa lesions in children, it has a few weaknesses. First, the diagnoses were made in a group of patients who were already aware of oral mucosa pathology or were referred to the clinic, for example by a pediatrician or orthodontist. In addition, the number and duration of lesions were not recorded, nor were general conditions or medications administered, that might have directly or indirectly affected the development of OMLs. Despite these limitations, the results of this research should be useful in providing more information about the frequency of oral mucosal lesions in pediatric population as well as their occurrence dependent on the gender and age of the child.

## 5. Conclusions

Our study provided observation of aspects and types of oral mucosa lesions prevalence in pediatric population aged 0–17 years. To the best of the authors’ knowledge, this is the first research of this type in Poland, as well as in Central Europe. The incidence of OMLs among children has been established at 5.21%. The most frequently observed pathology were aphthae. The majority of the patients were males and children in the age of 7–13 years (mean age of children was around 8 y/o). In boys, erosions and ulcers resulting from trauma were observed significantly more often. In preschoolers (0–6 y/o) geographic tongue was significantly more often diagnosed, while *Morsicatio buccarum* was significantly more often observed in school-aged children and adolescents (7–13, 14–17 y/o).

Knowledge about the prevalence of mucosal pathology in children is essential and fundamental for the medical practitioners for the appropriate diagnosis and treatment. They should be familiar not only with distinctive features of OMLs, which can sometimes be misleading, but also with their population incidence and with their frequency in connection to the age and gender of pediatric population, which is different from that of adults and the elderly. Further epidemiological studies are recommended for the child population in Europe for future preventive and health care service programs.

## Figures and Tables

**Table 1:** Total sample description.

	Age Groups	Nr of Patients	Mean Age
**Entire Study** **Group**	0–17	143 100%	8.4 (SD 5.2)
	0–6 Preschool	55 38.46%	2.8 (SD 1.9)
	7–13 School	58 40.56%	10 (SD 1.8)
	14–17 Adolescent	30 20.98%	15.5 (SD 1.2)
**Girls**	0–17	60 42%	8.6 (SD 5.1)
	0–6 Preschool	20 36.36%	2.7 (SD 1.9)
	7–13 School	27 46.55%	9.6 (SD 1.8)
	14–17 Adolescent	13 43.33%	15.7 (SD 1.0)
**Boys**	0–17	83 58%	8.2 (SD 5.3)
	0–6 Preschool	35 63.64%	2.9 (SD 1.9)
	7–13 School	31 53.45%	10.5 (SD 1.7)
	14–17 Adolescent	17 56.67%	15.4 (SD 1.2)

No statistically significant differences in the distribution of gender and age.

**Table 2:** All of the diagnosed oral mucosa lesions.

		Entire Study Group				Girls				Boys			
	Diagnosis	0–6 *n* = 55	7–13 *n* = 58	14–17 *n* = 30	Σ *n* = 143	0–6 *n* = 20	7–13 *n* = 27	14–17 *n* = 13	Σ *n* = 60	0–6 *n* = 35	7–13 *n* = 31	14–17 *n* = 17	Σ *n* = 83
**1**	Aphthae	7	15	5	27	1	5	3	9	6	10	2	18
**2**	Mucocele	2	9	4	15	1	5	2	8	1	4	2	7
**3**	*Morsicatio buccarum*	2	5	6	13	0	0	3	3	2	5	3	10
**4**	Hairy tongue	5	3	2	10	0	2	0	2	5	1	2	8
**5**	Fibroma	4	2	3	9	1	1	2	4	3	1	1	5
**6**	Geographic tongue	7	1	0	8	3	1	0	4	4	0	0	4
**7**	Papilloma	3	3	1	7	3	2	0	5	0	1	1	2
**8**	Abnormal upper lip frenulum	0	4	2	6	0	4	0	4	0	0	2	2
**9**	Pyogenic granuloma	1	5	0	6	0	3	0	3	1	2	0	3
**10**	Traumatic erosions and ulcers	2	2	2	6	0	0	0	0	2	2	2	6
**11**	Gingival enlargement	0	3	2	5	0	1	1	2	0	2	1	3
**12**	Oral Candidiasis	2	1	1	4	2	1	1	4	0	0	0	0
**13**	Melanoplakia	3	0	0	3	2	0	0	2	1	0	0	1
**14**	Herpetic stomatitis	3	0	0	3	1	0	0	1	2	0	0	2
**15**	Gingival cyst	2	0	0	2	0	0	0	0	2	0	0	2
**16**	Ankyloglossia	2	0	0	2	2	0	0	2	0	0	0	0
**17**	Frictional hyperkeratosis	0	1	1	2	0	1	1	2	0	0	0	0
**18**	Eruptive cyst	2	0	0	2	1	0	0	1	1	0	0	1
**19**	*Linea alba*	0	0	1	1	0	0	0	0	0	0	1	1
**20**	Hack’s disease	0	1	0	1	0	0	0	0	0	1	0	1
**21**	Granulomatous cheilitis	0	1	0	1	0	0	0	0	0	1	0	1
**22**	Gingivitis	0	1	0	1	0	0	0	0	0	1	0	1
**23**	Mucosal atrophy	1	0	0	1	1	0	0	1	0	0	0	0
**24**	Pigmented nevus	1	0	0	1	0	0	0	0	1	0	0	1
**25**	Contact allergic reaction	1	0	0	1	0	0	0	0	1	0	0	1
**26**	Leukoedema	1	0	0	1	0	0	0	0	1	0	0	1
**27**	Epstein pearls	1	0	0	1	1	0	0	1	0	0	0	0
**28**	Bohn nodules	1	0	0	1	0	0	0	0	1	0	0	1
**29**	Bone exostosis	1	0	0	1	1	0	0	1	0	0	0	0
**30**	Exfoliative cheilitis	1	0	0	1	0	0	0	0	1	0	0	1
**31**	Macroglossia	0	1	0	1	0	1	0	1	0	0	0	0

**Table 3:** The 10 most frequent oral mucosa lesions (OMLs) in pediatric patients in relation with gender.

	Diagnosis	Entire Study Group	%	Girls *n* = 60	Girls 42%	Boys *n* = 83	Boys 58%	χ^2^	*p*
**1**	Aphthae	27	18.9	9	15.0	18	21.7	1.02	0.31
**2**	Mucocele	15	10.5	8	13.3	7	8.4	0.89	0.34
**3**	*Morsicatio buccarum*	13	9.1	3	5.0	10	12.0	2.09	0.15
**4**	Hairy tongue	10	7.0	2	3.3	8	9.6	2.13	0.14
**5**	Fibroma	9	6.3	4	6.7	5	6.0	0.07	0.79
**6**	Geographic tongue	8	5.6	4	6.7	4	4.8	0.22	0.63
**7**	Papilloma	7	4.9	5	8.3	2	2.4	2.62	0.10
**8**	Abnormal upper lip frenulum	6	4.2	4	6.7	2	2.4	1.57	0.21
**9**	Pyogenic granuloma	6	4.2	3	5.0	3	3.6	0.00	0.96
**10**	Traumatic erosions and ulcers	6	4.2	0	0.0	6	7.2	4.53	0.03

**Table 4:** The most frequent oral mucosa lesions (OMLs) in children in correlation with age groups.

	Diagnosis	0–6 *n* = 55	% 38.5%	7–13 *n* = 58	% 40.6%	14–17 *n* = 30	% 20.9%	Σ *n* = 143	χ^2^_2_	*p*
**1**	Aphthae	7	12.7	15	25.9	5	16.7	27	3.30	0.19
**2**	Mucocele	2	3.6	9	15.5	4	13.3	15	4.57	0.10
**3**	*Morsicatio buccarum*	2	3.6	5	8.6	6	20.0	13	6.32	0.04
**4**	Hairy tongue	5	9.1	3	5.2	2	6.7	10	0.673	0.71
**5**	Fibroma	4	7.3	2	3.4	3	10.0	9	1.61	0.45
**6**	Geographic tongue	7	12.7	1	1.7	0	0.0	8	8.72	0.01
**7**	Papilloma	3	5.5	3	5.2	1	3.3	7	0.204	0.90
**8**	Lip-tie	0	0.0	4	6.9	2	6.7	6	3.92	0.14
**9**	Pyogenic granuloma	1	1.8	5	8.6	0	0.0	6	3.46	0.18
**10**	Traumatic erosions and ulcers	2	3.6	2	3.4	2	6.7	6	0.579	0.75

## Data Availability

The data that support the findings of this study are available from the corresponding author, J.E.O.-D., upon reasonable request.
